# Editorial: Innate Immunity Pathways in Autoimmune Diseases

**DOI:** 10.3389/fimmu.2019.01245

**Published:** 2019-06-04

**Authors:** Moncef Zouali, Antonio La Cava

**Affiliations:** ^1^Graduate Institute of Biomedical Sciences, China Medical University, Taichung City, Taiwan; ^2^Department of Medicine, University of California, Los Angeles, Los Angeles, CA, United States

**Keywords:** toll-like receptor, dendritic cells, innate-like B cells, extracellular vesicle, semaphorin, lupus, arthritis, calcium-dependent peptidyl-arginine deiminase

The immune system has evolved to protect the organism against infectious threats and to prevent the emergence of tumors, while preserving homeostatic immune integrity. Moreover, to avoid destruction of its own tissues, the immune system undergoes developmentally ordered processes that create the basis of immune tolerance to self. Autoimmune diseases develop when regulatory mechanisms of self-tolerance become impaired and/or deregulated.

Since immune tolerance is not innate, but is established during fetal life and postnatal development through an array of sophisticated mechanisms, unraveling the pathways that underlie the generation of pathogenic autoimmune reactions has typically focused on the adaptive branch of the immune system. Recently, however, it has been demonstrated that innate immunity can also play important roles in the initiation and/or progression of autoimmune disease. This Research Topic provides discussions of how innate immunity influences autoimmune diseases.

## Innate-Like B Cells and Autoimmunity

The role of B-lymphocytes was initially believed to be limited to immunoglobulin secretion. With further study, it became evident that B-cells also engage in the innate branch of the immune system and can modulate the activity of other cell populations. As reviewed in this Research Topic, it is possible that targeting certain innate-like B cell subsets could represent a novel therapeutic approach for inducing resolution of inflammation of autoimmune and inflammatory responses Tsay and Zouali.

## Immune Cell-Derived Extracellular Vesicles

Virtually all cell types release nano- to micro-scale membrane-enclosed vesicles, termed extracellular vesicles (EVs). Their potential to mediate intercellular communications is triggering intense research in a variety of disciplines. In this Research Topic, immune cell-derived EVs are shown to efficiently shuttle a TLR3 ligand to synovial fibroblasts and to potentially activate proinflammatory responses, suggesting their involvement in the pathogenicity of rheumatoid arthritis (RA) Frank-Bertoncelj et al. Interestingly, investigations into an experimental model of primary progressive multiple sclerosis support their potential therapeutic usefulness (1).

## Overcoming Plasmacytoid Dendritic Cell Immune Tolerance

Plasmacytoid dendritic cells (pDCs) are key actors in antiviral and antitumor immunity. This highly specialized cell subset plays a central role at the interface of innate and adaptive immunity through secretion of type I interferons (IFNs) and expression of TLRs, but also receptors involved in preventing abnormal immune responses. Crosslinking of these regulatory receptors could therefore efficiently suppress abnormal cytokine production. Accordingly, studies reported in this Research Topic indicate that pharmacological targeting of MEK1/2-ERK signaling could be a strategy to overcome immune tolerance of pDCs and re-establish their immunogenic activity Janovec et al. However, as discussed in this Research Topic, the increased expression of type I IFNs-regulated genes (the so-called “type I IFN signature”) in RA patients could be heterogeneous and dependent on the clinical stage of the disease Rodriguez-Carrio et al. This observation suggests that clinical and therapeutic considerations have to be tailored according to the autoimmune patient's characteristics rather than to the disease itself.

## Modulating Functional Properties of TLR for Therapeutic Purposes

Homo- or hetero-dimerization of TLRs can be induced by agonist binding, which can lead to lateral translocation in the membrane until the recruitment of downstream adaptors. As discussed in this Research Topic, identification of TLR micro-domains that participate in membrane association and orientation of individual domains could be targeted using novel activators or inhibitors to modulate TLR functional properties and reach the desired therapeutic effects Patra et al.

## Chronotherapeutic Targeting of TLRs in Autoimmunity

In responses to drugs, genome-wide association studies are used to map and characterize genes that contribute to inter-individual variability. While, our understanding of how particular genes determine the dynamics of drug effects remains incomplete, chronotherapy aims to design schedules of drug intake based on circadian biorhythms, cyclic production of factors, and receptor expression on target cells (2). This Research Topic presents information that could be useful to design clinical studies aiming to achieve chronotherapeutic HCQ effects and to reduce the pathological consequence of TLR9 activation Martinez-Garcia et al.

## Epigenetic Insufficiency in an Auto-Inflammatory Milieu

In autoimmune diseases, gene expression based solely on DNA sequence alterations and/or mutations is not sufficient to explain the variety of clinical manifestations observed, indicating that epigenetic deregulation contributes to the severity of these disorders. For example, there is an altered expression of Dicer1 (an endoribonuclease involved in the maturation of small non-coding RNAs such as microRNAs) in experimental mice (3), and β-cell specific disruption of Dicer1 leads to progressive impairment of insulin secretion and development of diabetes mellitus (4). As discussed in this Research Topic, reduced Dicer1 expression could contribute to a vicious cycle during which inflammation, together with inappropriate innate immunity responses, would create appropriate conditions for the initiation and/or progression of autoinflammatory diseases De Cauwer et al.

## Endogenous Regulators of the Inflammatory Response

Semaphorins comprise secreted and membrane-bound proteins endowed with immunomodulatory functions. Their potential relevance to autoimmune diseases comes from animal studies. For example, Semaphorin-3A (Sema3A) has beneficial therapeutic effects in a mouse model of RA. As discussed in this Research Topic, Sema3A was also efficient in both treating and preventing glomerular damage in a lupus mouse model Bejar et al. At present, its potential therapeutic usefulness remains uncertain because it can also induce autoimmune disease when overexpressed (5).

Other regulators of inflammatory responses are the calcium-dependent peptidyl-arginine deiminases (PADs) that citrullinate extracellular proteins during inflammation. In this Research Topic, active extracellular PADs are shown to play an important role in RA, a disease where affected joints have high levels of protein citrullination and autoantibodies against citrullinated proteins are important for its diagnosis Zhou et al.

Cytokines, such as IL-15, could represent new targets of innate immune response regulation, considering that its over-expression is shown in this Research Topic to reduce complement-dependent cytotoxicity and astrocyte loss in a mouse model of neuromyelitis optica Li et al.

## Conclusions

Mounting evidence has convincingly demonstrated that innate immune responses play a critical role in the initiation, progression, and maintenance of autoimmune disease. This Research Topic provides multiple examples of how different innate immunity components can contribute to the inflammatory processes that precede, underlie, and accompany the clinical manifestations of autoimmune disease. Further investigations have the potential to unveil new targets of therapeutic intervention that might ultimately lead to improved diagnosis, management, and outcomes in autoimmune disease patients.

## Author Contributions

All authors listed have made a substantial, direct and intellectual contribution to the work, and approved it for publication.

### Conflict of Interest Statement

The authors declare that the research was conducted in the absence of any commercial or financial relationships that could be construed as a potential conflict of interest.
